# Novel MicroRNA Reporter Uncovers Repression of Let-7 by GSK-3β

**DOI:** 10.1371/journal.pone.0066330

**Published:** 2013-06-26

**Authors:** Rong Guo, Kotb Abdelmohsen, Patrice J. Morin, Myriam Gorospe

**Affiliations:** 1 Laboratory of Genetics, National Institute on Aging-Intramural Research Program, NIH, Baltimore, Maryland, United States of America; 2 Laboratory of Molecular Biology and Immunology, National Institute on Aging-Intramural Research Program, NIH, Baltimore, Maryland, United States of America; 3 American Association for Cancer Research, Philadelphia, Pennsylvania, United States of America; University of Pennsylvania School of Medicine, United States of America

## Abstract

Several members of the let-7 microRNA family are downregulated in ovarian and other cancers. They are thought to act as tumor suppressors by lowering growth-promoting and anti-apoptotic proteins. In order to measure cellular let-7 levels systematically, we have developed a highly sensitive let-7 reporter assay system based on the expression of a chimeric mRNA that contains the luciferase coding region and a 3′-untranslated region (UTR) bearing two let-7-binding sites. In cells expressing the reporter construct, termed pmirGLO-let7, luciferase activity was high when let-7 was absent, while luciferase activity was low when let-7 levels were elevated. The ovarian cancer cell lines BG-1 and UCI-101 were transfected with the let-7 reporter and surveyed with a library of kinase inhibitors in order to identify pathways affecting let-7 activity. Among the inhibitors causing changes in endogenous let-7 abundance, the lowering of glycogen synthase kinase 3 (GSK-3)β function specifically increased let-7 levels and lowered luciferase activity. Similarly, silencing GSK-3β increased both mature and primary-let-7 levels in BG-1 cells, and decreased BG-1 cell survival. Further studies identified p53 as a downstream effector of the GSK-3β-mediated repression of let-7 biosynthesis. Our studies highlight GSK-3β as a novel therapeutic target in ovarian tumorigenesis.

## Introduction

Ovarian cancer is the fifth most common cancer among women and is the leading cause of death from gynecological cancers. The high mortality rate of ovarian cancer is partly due to the late-stage diagnosis and the high prevalence of drug resistance among ovarian cancer patients. Molecular markers for early screening and targeted therapy would thus permit more effective ways to combat this malignancy. MicroRNAs (miRNAs) are emerging as new diagnostic, prognostic, and therapeutic molecules in a number of disease processes including cancer (reviewed in [1]). These noncoding regulatory RNAs, spanning ∼22 nucleotides, have been implicated in key regulatory functions in many species. By binding to complementary sequences in target messenger RNAs (mRNAs), miRNAs post-transcriptionally regulate gene expression through translational repression and/or mRNA decay [2], [3]. Through their actions on collections of target mRNAs, miRNAs regulate diverse cellular processes relevant to cancer development and progression, such as cell differentiation, extracellular matrix remodeling, angiogenesis, proliferation, and apoptosis [4], [5].

Lethal-7 (let-7), one of the first tumor-suppressive miRNAs identified, is downregulated in ovarian cancer [6]. Let-7 was initially discovered as being essential for the timing of cell fate determination in *C. elegans* and is highly conserved across animal species [7]–[9]. Four out of the nine distinct let-7 loci in the human genome are in segments that are frequently deleted in human cancers [10]. Besides ovarian cancer, let-7 miRNAs are downregulated in numerous different cancers, including cancers of the lung, prostate, breast, and thyroid [11]–[14]. Low levels of let-7 correlate with poor prognosis, while overexpression of let-7 inhibits cancer growth in lung cancer and breast cancer mouse models [11], [15]–[17].

Let-7 can impair tumorigenesis by targeting oncoproteins like those in the Ras family and HMGA2 [18]–[25]. The transcripts encoding the three human Ras proteins (*NRAS*, *KRAS* and *HRAS* mRNAs), as well as *HMGA2* mRNA, all have let-7 binding sequences (named ‘let-7 complementary sites’ or LCS) in their 3′-untranslated regions (UTRs) which affect Ras and HMGA2 expression levels [18], [23]. Based on these studies, let-7 family members are believed to function as tumor suppressors. Accordingly, high Ras levels correlated with low let-7 levels in lung tumors but not in normal adjacent tissues, and HMGA2 acquired mutations that removed let-7 binding sites in tumors, thus avoiding let-7-mediated tumor suppression [18], [23].

Despite the clear inverse correlation between let-7 levels and cancer, the mechanisms that control let-7 abundance in tumors are poorly understood. Like other miRNAs, expression of the let-7 family is first regulated at the level of transcription, with the synthesis of a long primary (pri)-miRNA by RNA polymerase II. The pri-miRNA processing complex (the ‘microprocessor’), which includes the RNase III enzyme Drosha and its partner DGCR8, cleaves the pri-miRNA into 70-nt long precursor (pre)-miRNAs, which are transported to the cytoplasm by the nuclear export protein Exportin 5. Another RNase III enzyme, Dicer, subsequently cleaves pre-miRNA into the functional, ∼22-nt long mature miRNA. The mature miRNA binds argonaute (Ago) and other proteins to form the RNA-induced silencing complex (RISC), which specifically interacts with the target mRNA (reviewed in [26], [27]).

The transcriptional control of let-7 biosynthesis has already implicated numerous transcription factors, including c-Myc, p53, and NF-κB. c-Myc can bind to various enhancers of the let-7a1∼let-7d cluster (for eventual synthesis of let-7a1, let-7f1, and let-7d), and can both activate and inhibit cluster promoter activity, depending on enhancer activity [28], [29]. p53 binds directly to the let-7a3 and let-7b gene enhancer and represses let-7a and let-7b expression in HCT116 colon cancer cells in response to radiation and oxidative stress, while NF-κB induces expression from the let-7a/b endogenous promoter by binding to the basal let-7 promoter [30], [31]. The lack of correlation between the expression levels of pri-let-7 and mature let-7 miRNA observed sometimes suggests that let-7 is also subject to post-transcriptional regulation [8],[32],[33]. One of the best characterized post-transcriptional repressors of let-7 is the RNA-binding protein Lin28, which has two paralogs in mammalian cells, Lin28A and Lin28B. Lin28A is present in the cytoplasm and blocks Dicer processing of pre-let-7 by inducing terminal uridylation of pre-let-7, while Lin28B is nuclear and sequesters pri-let-7, blocking its nuclear processing [34], [35]. In addition, c-Myc can also post-transcriptionally control let-7 production by binding to the *LIN28B* promoter and inducing Lin28B expression [36].

In order to systematically investigate the mechanisms that control let-7 biogenesis, we sought to develop a fast screening method to quantify the cellular levels of this tumor suppressor miRNA. Here, we describe the development of a novel luciferase-based, highly sensitive reporter system that allows the rapid measurement of let-7 in cells. Using this construct we screened a kinase inhibitor library and discovered that GSK-3β repressed let-7 production in ovarian cancer cells, thus uncovering GSK-3β as a novel target for therapeutic intervention.

## Materials and Methods

### Cell Culture and Transfection

The ovarian cancer cell lines BG-1 and UCI-101 [37]–[40], as well as the colon cancer cell line HCT116 (p53+/+ and p53−/−) were cultured in McCoy’s 5A media supplemented with 10% fetal bovine serum and antibiotics. Cells were incubated at 37°C in an atmosphere of 5% CO_2_. For overexpression of let-7, BG-1 and UCI-101 cells were seeded at a density of 3×10^4^ cells per well in 24-well plates and transfected with let-7 precursors (Ambion) individually or with a negative control (Ctrl miRNA, Ambion) at a final concentration of 100 nM, using siPort NeoFX transfection agent (Ambion). Twenty-four hours later, cells were transfected with 50 ng of each luciferase vector per well using Lipofectamine 2000 (Invitrogen); 24 h after that, cells were lysed and firefly and renilla luciferase activities were measured using a Dual-luciferase assay system (Promega) and a 96-well Microplate Luminometer BD Pharmingen™ Monolight 3096. The ratio of firefly luciferase (FL) activity to renilla luciferase (RL) activity was determined. All values were then normalized to those of Ctrl miRNA-transfected cells. To knock down GSK-3β, BG-1 cells were seeded in 12-well plates at a density of 7×10^4^ cells per well. The next day, cells were transfected with GSK-3β siRNA, p53 siRNA or control (Ctrl) siRNA (all from Santa Cruz Biotechnology; final concentration 100 nM) using X-tremeGENE siRNA Transfection Reagent (Roche).

### Kinase Inhibitor Library Assay

BG-1 and UCI-101 cells were seeded at a density of 5,000 cells per well in black well/clear bottom, Optilux 96-well plates and transfected with reporter plasmid as explained above. Five hours later, the transfection media was removed, changed to regular culture media, and supplemented with the kinase inhibitor library (Enzo Life Sciences) at a final concentration of 10 µM; vehicle DMSO was included as negative control. Twenty-four hours after treatment, luciferase activity was measured and calculated as explained above. All of the readings were normalized to DMSO readings.

### Cytotoxicity Assays

BG-1 cells were seeded in 6-well plates at a density of 1.5×10^5^ cells per well; 24 h later, cells were transfected with 100 nM GSK-3β siRNA or Ctrl siRNA. To measure cell survival, 48 h after transfection, cells were trypsinized and counted using a hemocytometer. To measure cell death, floating cells were counted using a hemocytometer. All cell numbers were compared with those in with Ctrl siRNA-treated cells.

### Western Blot Analysis

Forty-eight h after transfection, protein samples prepared in lysis buffer (156 mM Tris-HCl, pH 6.8, 5% SDS, 25% glycerol) were mixed with 4× loading buffer (200 mM Tris-HCl, pH 6.8, 400 mM DTT, 8% SDS, 40% glycerol, and 0.4% bromophenol blue) and incubated for 5 min at 95°C. Primary antibodies recognizing GSK-3β and p53 were from Santa Cruz (used at 1∶500 dilution). Horseradish peroxidase-linked sheep secondary antibodies anti-rabbit and anti-mouse immunoglobulin (GE healthcare) were used at 1∶10,000. Signals were detected by enhanced chemiluminescence.

### RNA Analysis

Following transfection, total RNA was isolated using TRIzol according to the manufacturer’s protocol (Invitrogen). Total RNA was treated with DNase I (Qiagen) to eliminate DNA contamination.

To measure mature microRNA levels, 1 µg of total RNA was used to generate cDNA using TaqMan MicroRNA reverse transcription kit (Applied Biosystems) followed by TaqMan Universal Master Mix II and TaqMan microRNA assays according to the manufacturer’s protocol (Applied Biosystems). *U6* was measured as a control RNA. To measure primary microRNA levels, 100 ng total RNA was used to generate cDNA using Maxima Reverse Transcriptase (Thermo Scientific) followed by TaqMan Universal Master Mix II and TaqMan Pri-miRNA Assays according to the manufacturer’s protocol (Applied Biosystems). *18S* rRNA was measured using KAPA SYBR mix (Kapa Biosystems) as the loading control. All PCR was performed in triplicate using an Applied Biosystems 7300 Real Time PCR System.

For pri-mRNA stability analysis, BG-1 cells were treated with 2 µg/ml actinomycin D for the times shown, whereupon the levels of pri-let-7 transcripts in each transfection group were measured by RT-qPCR from total RNA using Taqman let-7-specific primer pairs. *GAPDH* mRNA was measured as a stable control transcript that encodes a housekeeping protein. The levels of pri-let-7 and *GAPDH* mRNAs were normalized to *18S* rRNA levels and plotted as the percentage of mRNA remaining compared with the levels of the same mRNA at time zero.

### Luciferase Reporter Constructs

LCS 2-8 (1560 bp, 1784-3244) and LCS 2-6 (756 bp, 1784-2540) from the 3′ UTR of human *KRAS* variant 2 (NM_004985.3), LCS 2-8 (1475 bp, 1088-2563) and LCS 2-5 (600 bp, 1088-1688) from human *HMGA2* variant 1 (NM_003483) were amplified using the following primer sets: RASF1 and RASR1, RASF1 and RASR2, HMGAF1 and HMGAR1, HMGAF1 and HMGAR2. The PCR products were cloned into the dual-luciferase vector pmirGLO (Promega) using SacI and XhoI sites to generate pmirGLO-KRAS2-8 and -2-6, and pmirGLO-HMGA2-8 and -2-5. Two sets of primers (Let7fF1 with Let7fR1, and Let7fF2 with Let7fR2) were annealed together and cloned into pmirGLO SacI and XhoI sites to generate pmirGLO-let7 and pmirGLO-let7(s).

### Primers

RASF1AAAGAGCTCGTTTTAGGACTCTTCTTCCATATTAG

RASF1AAAACTCGAGGCCCATCTACATCAAAAATTC

RASF2AAAACTCGAGTTCTGCAAAACAGGTTTATG

HMGAF1AAAGAGCTCGAAAGACCTGAATACCAC

HMGAF1AAAACTCGAGTTTTCAGTATGAAAAATGAGG

HMGAF2AAAACTCGAGCCTTTTGAAGTCTGG

Let7fF1P-CAACTATACAATCTACTACCTCAACGCGATGTAAATATCGCAATCCCTTTAACTATACAATCTACTACCTCAC

Let7fF1P-TCGAGTGAGGTAGTAGATTGTATAGTTAAAGGGATTGCGATATTTACATCGCGTTGAGGTAGTAGATTGTATAGTTGAGCT

Let7fF2P-CAACTATACAATCTACTACCTCAATCGTAGCACAACTATACAATCTACTACCTCAC

Let7fF2P-TCGAGTGAGGTAGTAGATTGTATAGTTGTGCTACGATTGAGGTAGTAGATTGTATAGTTGAGCT

## Results

### Novel microRNA Reporter to Measure let-7 Function in vivo

The pmirGLO Vector (Promega), containing firefly luciferase (luc2) as primary reporter gene and renilla luciferase (hRluc) as an internal control reporter for normalization, was chosen to quantify miRNA activity. After inserting miRNA target sites into the 3′ end of the firefly luciferase gene, reduced firefly luciferase (FL) activity was predicted to be an accurate and sensitive read-out of the interaction of a miRNA present in the cell with the cloned miRNA target sequence. The set of test reporters prepared and assayed included two vectors that contained let-7 target sites (LCS) from human *KRAS* 3′UTR (pmirGLO-KRAS2-8 and -2-6), two that included LCS from *HMGA2* 3′UTR (pmirGLO-HMGA2-8 and -2-5), and two vectors containing two copies of let-7f complementary sequences (AACUAUACAAUCUACUACCUCAA) separated by a 27-nucleotide spacer from lin-41 3′UTR (ACGCGAUGUAAAUAUCGCAAUCCCUUU) [41] (pmirGLO-let7f-lin41, hereafter pmirGLO-let7) or by 10 random nucleotides [pmirGLO-let7f-10random, hereafter pmirGLO-let7(s)] (Figure S1). The reporters, along with an empty control vector, were transfected into two ovarian cancer cell lines, UCI-101 and BG-1, which expressed different endogenous levels of let-7 (Figure S2A). Following transfection of either a precursor RNA for let-7f (pre-let-7f) or control RNA (Ctrl miRNA) into BG-1 or UCI-101 cells, the plasmids bearing the two let-7 sites separated by 27 or 10 nucleotides pmirGLO-let7 and pmirGLO-let7(s), respectively, exhibited the highest levels of repression (Figure S2B,C) and were therefore deemed most sensitive. Accordingly, pmirGLO-let7 was selected for further experiments.

**Figure 1:**
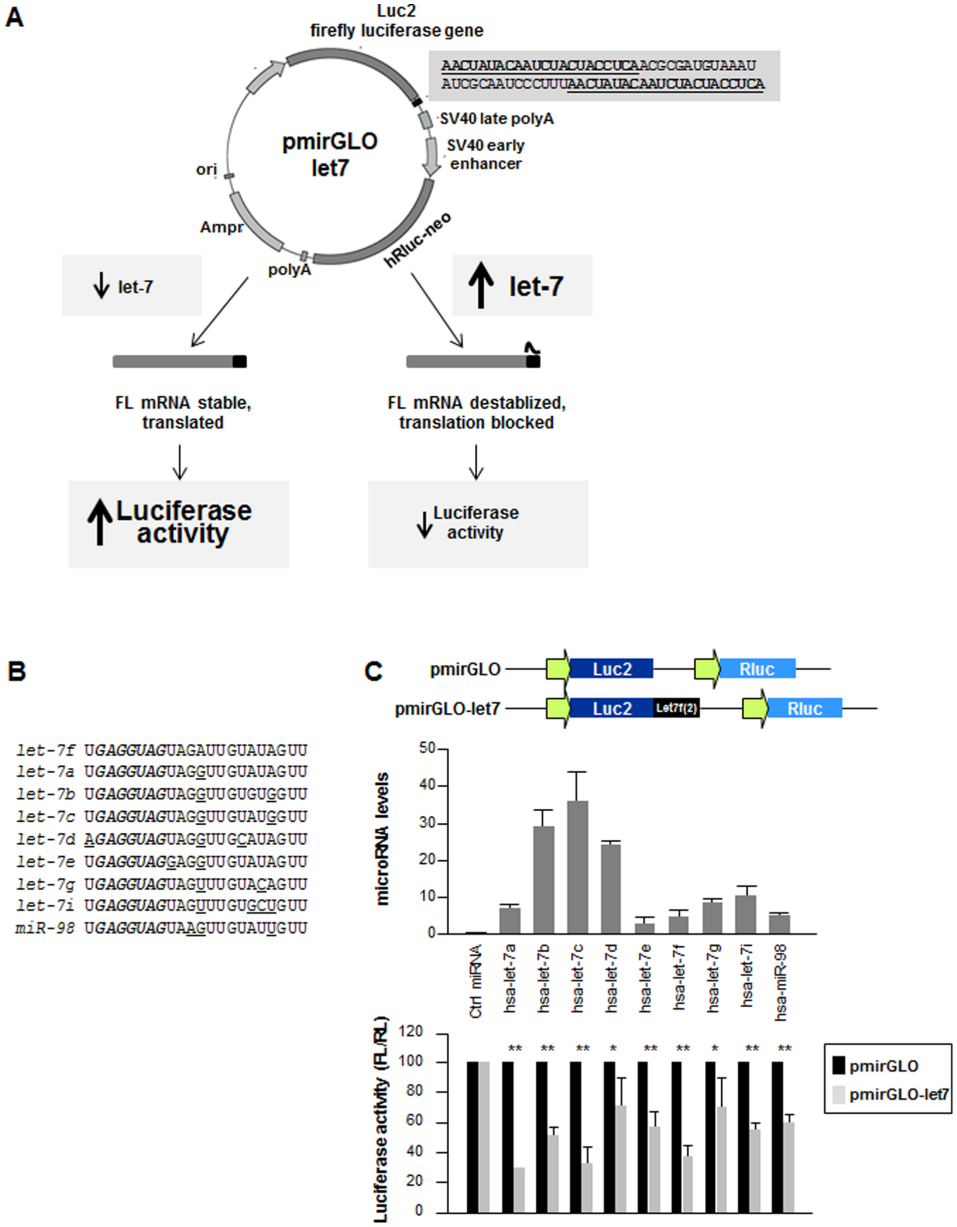
Novel luciferase reporter pmirGLO-let7 to measure let-7 levels in cells. (**A**) Schematic of pmirGLO-let7 vector (adapted from Promega), a dual luciferase vector containing two let-7 target sites (underlined nucleotides, with a 27-nt spacer from lin41, Figure S1). (**B**) Mature sequences of the highly conserved let-7 family; sequence in bold (nucleotides 2–8) are the shared seed region and underlined nucleotides vary between let-7f and other let-7 miRNAs. (**C**) Forty-eight hours after transfecting BG-1 cells with 100 nM let-7 family precursors or with control (Ctrl) miRNA, total RNA was collected and the overexpressed microRNAs were measured by RT-qPCR analysis (*top*). Twenty-four hours after transfection as described in the top panel, BG-1 cells were transfected with pmirGLO-let7; 24 h after that, luciferase activity was measured, normalized to luciferase activity in Ctrl miRNA. Data are the means and standard deviation (S.D.) from three independent experiments: * *P*<0.05; ** *P*<0.01.

There are 9 major forms of the mature human let-7. All of them are highly conserved and share the ‘seed’ region of high complementarity with the target mRNA that is crucial for miRNA targeting and function (Figure 1B). Precursors of the 9 let-7 miRNAs (let-7a, let-7b, let-7c, let-7d, let-7e, let-7f, let-7g, let-7i and miR-98) were transfected individually into BG-1 cells and transfection was confirmed 24 h later by reverse transcription (RT) followed by real-time, quantitative (q)PCR analysis using TaqMan® probes (Figure 1C, *top* graph). pmirGLO-let7 was then transfected into BG-1 cells and luciferase activity (FL/RL) was measured 24 h after that. As shown in Figure 1C (*bottom* graph), all of the different let-7 variants repressed luciferase activity significantly in BG-1 cells, indicating that pmirGLO-let7 was an effective reporter to monitor overall let-7 levels in the cell.

### GSK-3β Represses let-7 Production in Ovarian Cancer Cells

Using pmirGLO-let7 as a reporter construct, we investigated the signaling pathways implicated in the synthesis of let-7 in ovarian cancer cells. We employed the Screen-Well™ Kinase Inhibitor Library (from Enzo Life Sciences), containing 80 well-characterized compounds capable of inhibiting a range of major kinases, including PI-3Kinase, CaM kinase II, JAK, PKA, CDK, JNK, PKC, CKI II, MAPK, RAF, EGFR, MEK, SAPK, GSK, MLCK, SRC, IKK, PDGFR, and VEGFR (Table S1). The library was used to determine which kinase or signaling pathway might be involved in upstream regulation of let-7 activity in ovarian cancer cells. BG-1 cells and UCI-101 cells were transfected with pmirGLO-let7, incubated with the kinase inhibitor library (10 µM final concentration) or vehicle (DMSO), and lysed for measurement of luciferase activity (FL/RL) 24 h later. Although the majority of compounds did not have any effect on reporter activity, a subset of drugs (Ro 31-8220, Kenpaullone) lowered luciferase levels significantly, suggesting that let-7 was elevated, and another subset of drugs (Genistein**,** Daidzein, Indirubin, Indirubin-3′-monoxime, SU 4312, AG-1296, Tyrphostin AG 1295, and Apigenin) increased luciferase activity, suggesting that let-7 levels were reduced (Table 1). In control experiments, these compounds were tested for their influence on two luciferase vectors (psiCHECK2-POLE4 and psiCHECK2-MKK4) which bear long 3′UTRs with sites for other microRNAs (miR-15b, miR-24, miR-25, and miR-141 for psiCHECK2-MKK4, and miR-519 for psiCHECK2-POLE4 [42], [43]) but not let-7; neither of these two luciferase reporter vectors detected endogenous let-7 activity (Figure S3) nor were they sensitive to the inhibitor compounds that affected pmirGLO-let7 (Tables S1 and S2).

**Table 1:** Survey of let-7 levels using a kinase inhibitor library.

Effect on pmirGLO-let7	Kinase inhibitors	Luciferase activity
Downregulatedreporter activity	**Ro 31-8220**	0.38
	**Kenpaullone**	0.51
No effect on reporter activity	PD-98059, U-0126, SB-203580, H-7, H-9, Staurosporine, AG-494, AG-825, Lavendustin A, RG-14620, Tyrphostin 23, Tyrphostin 25, Tyrphostin 46, Tyrphostin 47, Tyrphostin 51, Tyrphostin 1, Tyrphostin AG 1288, Tyrphostin AG 1478, Tyrphostin 9, HNMPA (Hydroxy-2-naphthalenylmethylphosphonic acid), PKC-412, Piceatannol, PP1, AG-490, AG-126, AG-370, AG-879, LY 294002, Wortmannin, GF 109203X, Hypericin, Sphingosine, H-89, H-8, HA-1004, HA-1077, HDBA (2-Hydroxy-5-(2,5-dihydroxybenzylamino)benzoic acid), KN-62, KN-93, ML-7, ML-9, 2-Aminopurine, N9-Isopropyl-olomoucine, Olomoucine, iso-Olomoucine, Roscovitine, 5-Iodotubercidin, LFM-A13, SB-202190, PP2, ZM 336372, GW 5074, Palmitoyl-DL-carnitine Cl, Rottlerin, Erbstatin analog, Quercetin dehydrate, SU1498, ZM 449829, BAY 11-7082, DRB (5,6-Dichloro-1-b-D-ribofuranosylbenzimidazole), HBDDE (2,2′,3,3′,4,4′-Hexahydroxy-1,1′-biphenyl-6,6′-dimethanol dimethyl ether), SP 600125, Y-27632, Terreic acid, Triciribine, BML-257, SC-514, BML-259, BML-265(Erlotinib analog), Rapamycin	0.60–1.80
Upregulated reporter activity	**Genistein**	1.99
	**Daidzein**	2.18
	**Indirubin**	1.86
	**Indirubin-3′-monoxime**	1.80
	**SU 4312**	2.14
	**AG-1296**	2.15
	**Tyrphostin AG 1295**	2.51
	**Apigenin**	1.80

Five hours after transfection of pmirGLO-let7, BG-1 cells were treated with the kinase inhibitor library and luciferase activity (FL/RL) was measured 24 h later. Inhibitor drugs causing ‘Downregulated reporter activity’ (elevated let-7) were those yielding luciferase activities <0.6, while drugs causing ‘Upregulated reporter activity’ yielded luciferase activities >1.8. All other drugs were classified as having ‘No effect on reporter activity’.

**Figure 2:**
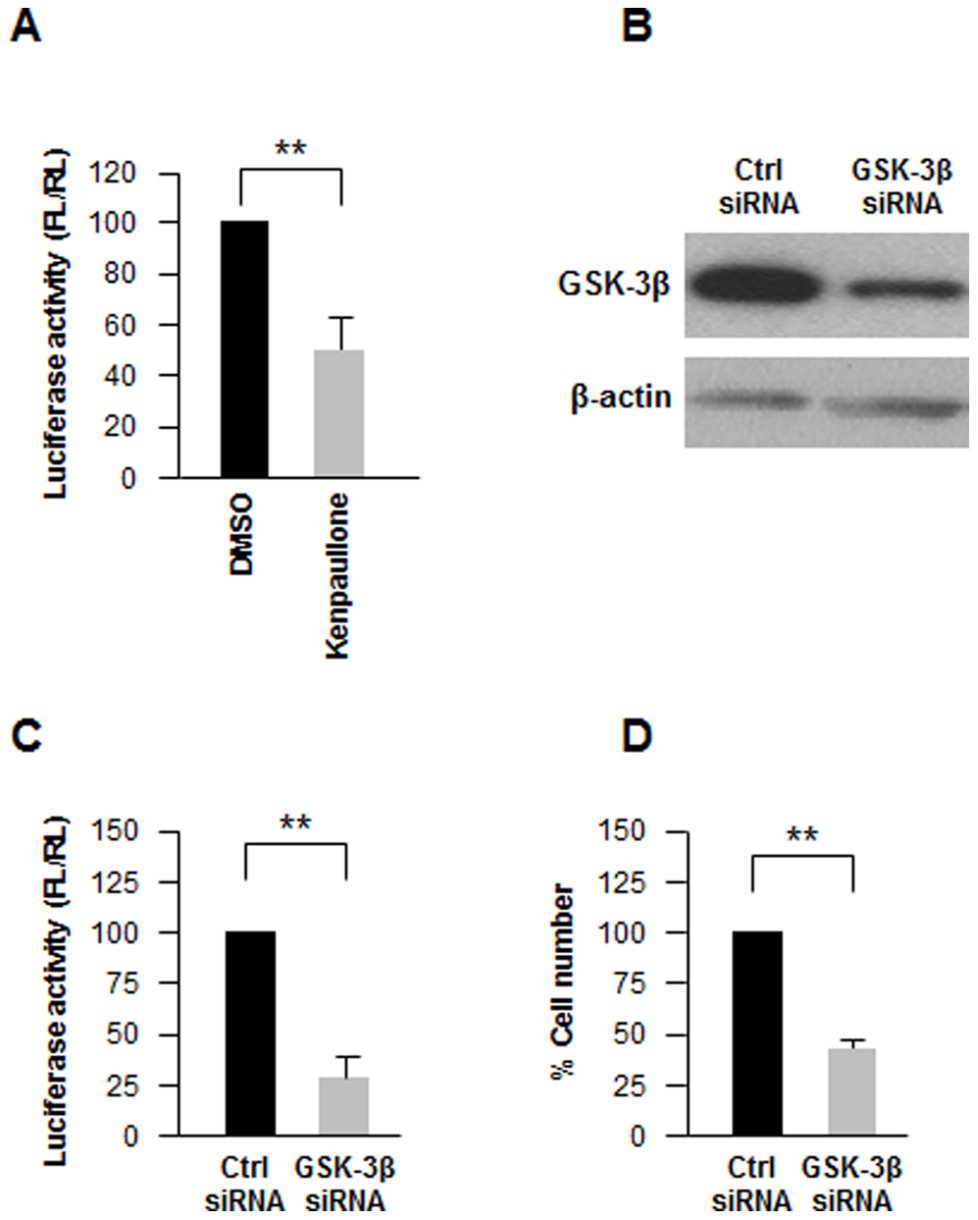
GSK-3β inhibition or silencing decreases reporter activity. (**A**) Five hours after transfection with pmirGLO-let7, BG-1 cells were treated with GSK-3β inhibitor Kenpaullone (10 µM) or with vehicle control (DMSO) and luciferase activity was measured 24 h later. (**B**) Forty-eight hours after transfecting BG-1 cells with 100 nM Ctrl siRNA or GSK-3β siRNA, lysates were prepared to assess the levels of GSK-3β or loading control β-actin by western blot analysis. (**C**) Twenty-four hours after transfection with 100 nM Ctrl siRNA or GSK-3β siRNA, BG-1 cells were transfected with pmirGLO-let7 and luciferase activity was measured 24 h later. (**D**) Forty-eight hours after transfection with 100 nM Ctrl siRNA or GSK-3β siRNA, BG-1 cells were trypsinized and live cells were counted. Data in (A, C and D) are the means of three independent experiments: ** *P*<0.01.

**Figure 3:**
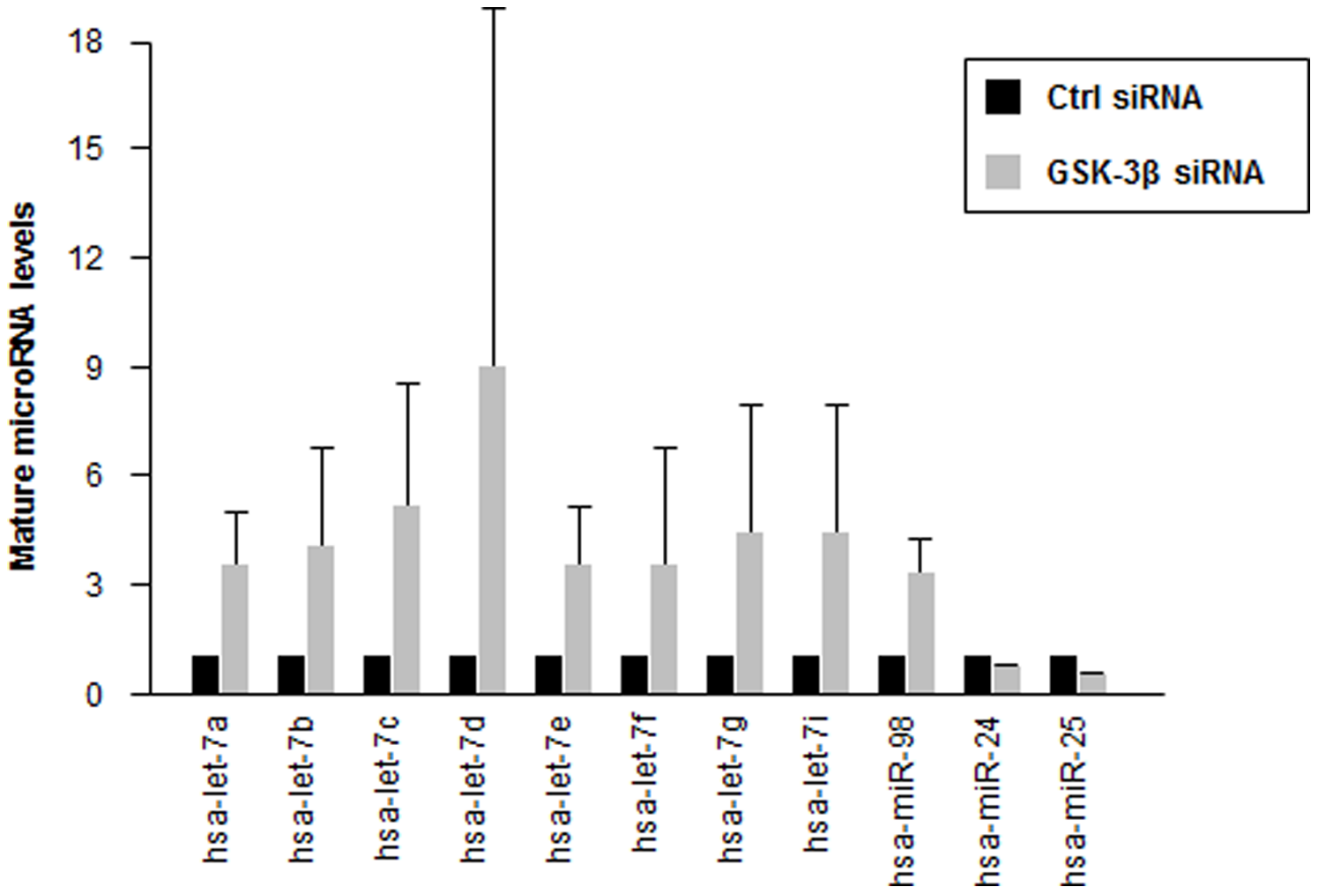
GSK-3β silencing increases mature let-7 levels. Forty-eight hours after transfecting BG-1 cells with 100 nM Ctrl siRNA or GSK-3β siRNA, total RNA was collected and mature let-7 miRNAs, as well as two control microRNAs (mir-24, and mir-25) were quantified by RT-qPCR and normalized to U6 snRNA. Data are the means and S.D. from at least three independent experiments.

Among the compounds that altered pmirGLO-let7 luciferase activity, the effect of kenpaullone, an inhibitor of GSK-3β [44], was confirmed after treating cells separately with this drug (Figure 2A). GSK-3β is an important enzyme in diverse signaling pathways involved in the regulation of glycogen metabolism, cell mobility, proliferation, cell survival, and differentiation, but its role in carcinogenesis remains controversial. Since kenpaullone, and in general the drugs in our screen, were not selective inhibitors of a single kinase, we sought further insight into the function of GSK-3β as regulator of let-7 expression by knocking down GSK-3β in BG-1 cells using small interfering (si)RNA (Figure 2B). As shown in Figure 2C, silencing GSK-3β in BG-1 cells potently reduced luciferase activity, in keeping with the effects of the inhibitor kenpaullone (Figure 2A). By contrast, luciferase activity in cells transfected with psiCHECK2-MKK4 or psiCHECK2-POLE4 was not lowered upon GSK-3β silencing (instead, it was modestly upregulated, Figure S4). Moreover, silencing GSK-3β lowered BG-1 cell survival, yielding 60% cell death by 48 h after transfecting cells with GSK-3β siRNA (Figure 2D). In keeping with the luciferase reporter results, silencing GSK-3β broadly increased the levels of let-7 in BG-1 cells, as measured by RT-qPCR analysis (Figure 3). This increase appeared to be specific, since silencing GSK-3β did not increase the levels of two other miRNAs (miR-24, miR-25). Together, these data indicate that GSK-3β can specifically reduce let-7 levels.

### GSK-3β Lowers let-7 Expression by Suppressing p53 Levels

**Figure 4:**
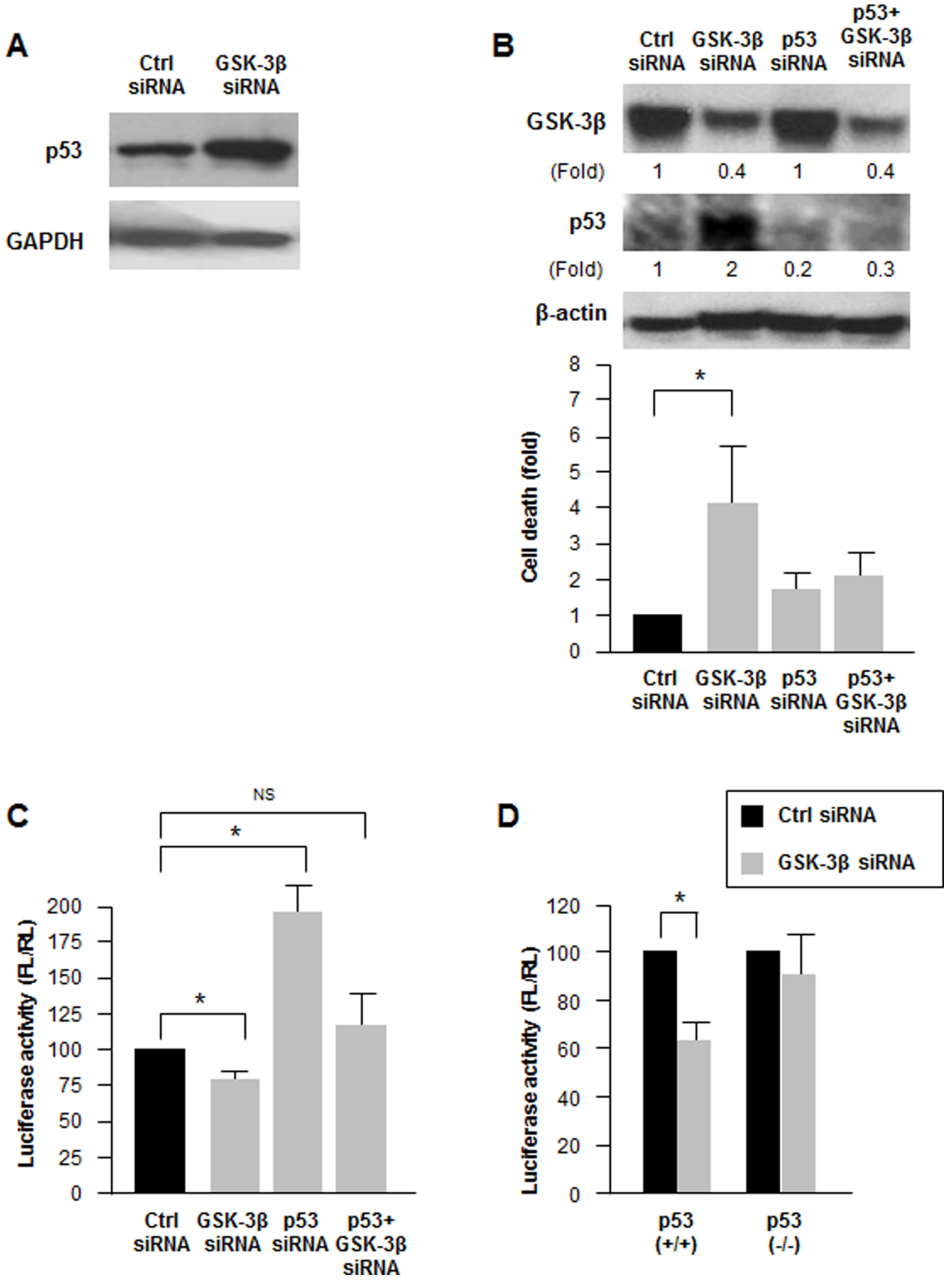
p53 is required for GSK-3β regulation on let-7 activity. (**A**) Forty-eight hours after transfecting BG-1 cells with Ctrl or GSK-3β siRNA as explained in Figure 2B, lysates were prepared to assess the levels of p53 and loading control GAPDH by western blot analysis. (**B**) Forty-eight hours after transfecting BG-1 cells with 50 nM Ctrl siRNA, GSK-3β siRNA, and/or p53 siRNA, lysates were prepared to measure protein levels (*top*) and cells in the culture media were collected and counted (*graph*). Signals were quantified by densitometry and normalized to the levels of β-actin (Fold). (**C**) Twenty-four hours after transfection as described in panel (B), BG-1 cells were transfected with pmirGLO-let7 and luciferase activity was measured 24 h later. (**D**) Twenty-four hours after transfection with Ctrl or GSK-3β siRNAs, HCT116 p53+/+ and p53−/− cells were transfected with pmirGLO-let7 and luciferase activity was measured 24 h later. Data in (B, C and D) are the means and S.D. from three independent experiments: * *P*<0.05; NS, not significant.

To investigate the downstream effectors of GSK-3β-mediated inhibition of let-7, we focused on evidence that silencing GSK-3β triggered cell death and elevated p53 expression in colon cancer cells [45]. We observed a similar effect in BG-1 cells, where silencing GSK-3β (as in Figure 2B) also increased p53 levels (Figure 4A) and where the cell death seen after silencing GSK-3β was partially rescued by simultaneously silencing p53 (Figure 4B). The notion that p53 was a downstream effector of the GSK-3β actions on let-7 abundance was supported by evidence that the loss in luciferase activity seen after GSK-3β silencing was rescued by silencing p53 (Figure 4C). Further support for the view that GSK-3β controls let-7 levels in a p53-dependent manner came from analysis of the colon cancer cell line HCT116, where wild-type cells (wt, expressing p53) and p53-null cells (obtained by somatic deletion of both p53 alleles [46]) were analyzed. As shown in Figure 4D, only wt cells [HCT116(+/+)] showed lower luciferase reporter levels after silencing GSK-3β, while p53-null cells [HCT116(−/−)] did not; the steady-state levels of let-7 were comparable in both cell lines (Figure S5). Collectively, these results support the notion that GSK-3β represses let-7 production by reducing the abundance of p53, a positive regulator of let-7 transcription.

### GSK-3β Silencing Increases pri-let-7 Levels, but not Stability

**Figure 5:**
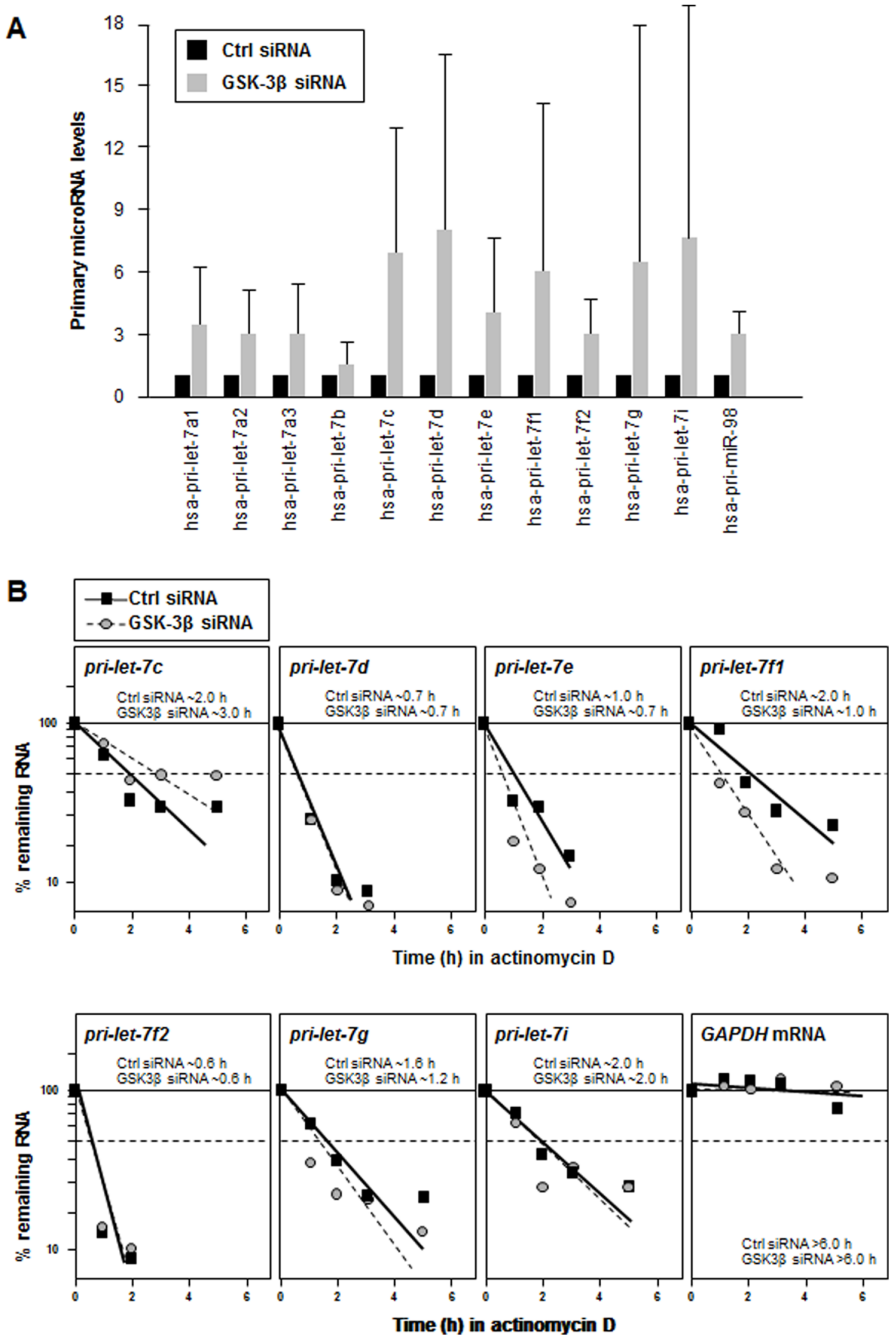
Pri-let-7 levels increase by GSK-3β silencing mainly via transcriptional upregulation. (**A**) Forty-eight hours after transfecting BG-1 cells with 100 nM Ctrl siRNA or GSK-3β siRNA, total RNA was collected and pri-let-7 levels were quantified by RT-qPCR and normalized to 18S rRNA. (**B**) Forty-eight hours after BG-1 cells were transfected as in (A), actinomycin D was added and RNA was collected at the times indicated for RT-qPCR analysis of the levels of pri-let-7 transcripts and *GAPDH* mRNAs. After normalizing to 18S rRNA levels, the half-life of each RNA was calculated as the time needed to reach 50% of its original abundance. Data are the average of two independent experiments showing similar results.

Since GSK-3β reduces let-7 production by lowering p53, and p53 was reported to control the transcriptional levels of several let-7 family members [47], we asked if GSK-3β might affect let-7 transcription. First, we tested this possibility by monitoring the levels of primary let-7 transcripts. As shown in Figure 5A, all of the pri-let-7 transcripts examined were upregulated upon GSK-3β silencing, suggesting that indeed the production of pri-let-7 transcripts (and not their subsequent processing into mature let-7) was the step most strongly regulated. Second, we tested if GSK-3β silencing altered instead the stability of pri-let-7 transcripts, which would ultimately change the abundance of mature let-7. To measure the half-lives of pri-let-7 transcripts, BG-1 cells expressing normal or silenced GSK-3β were treated with the drug actinomycin D, which blocks *de novo* transcription. Forty-eight hours later, the half-lives of pri-let-7 RNAs were assessed by measuring the time required for each pri-let-7 RNA to be reduced to 50% of their original abundance. As shown in Figure 5B, each pri-let-7 RNA had a distinct half-life; silencing GSK-3β actually increased the half-life of pri-let-7c, but the majority of pri-let-7 transcripts showed either unchanged (pri-let7d, -f2, -i) or lower stabilities (pri-let-7e, -f1, -g). As anticipated, the half-life of *GAPDH* mRNA, encoding a housekeeping protein, was not influenced by GSK-3β silencing. In sum, these results support the notion that the influence of GSK-3β on let-7 expression is strongly elicited at the transcriptional level, at least part of which is mediated by the transcription factor p53.

## Discussion

We have introduced a novel reporter construct (pmirGLO-let7) that permits the rapid screening of let-7 levels in a given cell population. Among the reporter constructs we examined, pmirGLO-let7, containing two let-7 sites separated by 27 nucleotides, as well as pmirGLO-let7(s), which only differed in having the let-7 sites separated by a shorter (10-nt) spacer, showed the highest sensitivity to changes in let-7 levels. Other reporters with more complex 3′UTRs were less responsive to let-7, likely due to the presence of other regulatory sites on the 3′UTR which masked the relative influence of let-7 (Figure S1). Under a variety of conditions, pmirGLO-let7 was found to permit the rapid screen of let-7 levels, or perhaps a more valuable parameter: ‘let-7 available for repression’.

Using one of the two more responsive reporter constructs, pmirGLO-let7, we screened a library of inhibitor compounds in order to identify kinases which influenced let-7 abundance in BG-1 cells. This analysis revealed that a GSK-3β inhibitor, kenpaullone, lowered luciferase activity, a reflection of higher let-7 levels (Table 1, Figure 2A). Since Kenpaullone was not a specific inhibitor of GSK-3β (it can also inhibit, at higher concentrations, kinases lck, cdk1, cdk2, cdk5, c-Src, casein kinase II, ERK1, and ERK2), we asked if GSK-3β controlled let-7 levels by silencing GSK-3β. Indeed, these experiments recapitulated the effects of the drug, as lowering GSK-3β expression similarly lowered luciferase activity and elevated the levels of all mature forms of let-7 (Figs. 2C and 3). Moreover, GSK-3β appeared to modulate mature let-7 levels mainly by controlling the transcription of the different let-7 genes, since the levels of the primary transcripts (pri-let-7s) were also selectively upregulated after silencing GSK-3β, and the differential stability of the pri-let-7 transcripts did not explain the increase in mature let-7 levels.

The precise transcriptional mechanisms through which GSK-3β represses let-7 levels are not fully understood. However, among the different transcription factors that are downstream effectors of GSK-3β – prominently β-catenin, although it is frequently undetectable or very low in ovarian cancer cells [48] – we identified p53 as a regulator of let-7 transcription. p53 had been shown to regulate the transcription of several let-7 variants [47] and was proposed to be a potential transcriptional regulator of other let-7 variants [49]. Interestingly, we found that GSK-3β silencing upregulated all let-7 species (Figure 3), although we did not fully examine if p53 was responsible for each of these regulatory events.

How p53 levels rise after silencing GSK-3β also remains to be studied in detail. Indeed, the mechanisms whereby GSK-3β regulate p53 expression and function are still controversial. While some evidence supports a role for GSK-3β as an inducer of p53 levels [50], there are numerous examples of GSK-3β suppressing p53 levels. For instance, endoplasmic reticulum (ER) stress enhanced GSK-3β function, which then led to p53 phosphorylation on Ser 376 (pS376), accelerating p53 degradation [51], [52]. Similarly, lowering GSK-3β activity increased p53 function in colorectal cancer cells linked to apoptosis and tumor suppression [45], while GSK-3β-mediated phosphorylation of Mdm2, a protein required for p53 degradation, triggered the rapid degradation of p53 [53]. In the system studied here, perhaps silencing GSK-3β blocked the Mdm2-elicited decay of p53, allowing p53 to accumulate; this possibility as well as other mechanisms of p53 upregulation, await further investigation.

Since GSK-3β is a pleiotropic kinase with a broad range of functions, it also appears to have multiple types of impact on carcinogenesis, with evidence supporting its tumorigenic influence and other evidence suggesting that it can inhibit tumor growth [54]–[62]. Our results support a role for GSK-3β in promoting cell survival, since silencing GSK-3β increased p53 expression and elevated cell death, and therefore a pro-tumorigenic influence. In this regard, pharmaceutical inhibitors of GSK-3β may have therapeutic benefit in ovarian cancer. Whether the effects of GSK-3β inhibition on cell survival are dependent, at least in part, on the upregulation of let-7 levels remains to be investigated. Likewise, the specific let-7 targets involved in the growth reduction in BG-1 cells await identification. It also remains to be studied whether GSK-3β regulates acute changes in let-7 levels, perhaps in response to stimuli that induce or inhibit GSK-3β function.

Finally, p53 and let-7 are well-known enhancers of cellular senescence. In this context, the discovery that inhibition of GSK-3β also brings about cellular senescence [52] raises the possibility that the reduction in cell number seen by lowering GSK-3β is mediated at least in part through the induction of p53 and let-7. The tools introduced in this report will help with the investigation of let-7 levels and the pathways that control its rich biological actions in different physiologic and pathologic settings.

## Supporting Information

Figure S1 **Construction of luciferase reporters.** **(A)** The human *KRAS* 3′UTR has 8 let-7 complementary sequences (LCS) that can interact with let-7f, while the human *HMGA2* 3′UTR has 8 copies of LCS interacting with let-7f. The interactions are indicated (blue text). Various sequences were inserted into pmirGLO vector multiple cloning site, including two regions from human *KRAS* 3′UTR (1560 bp KRAS 2-8 and 756 bp KRAS 2–6), and two regions from human *HMGA2* 3′UTR (1475 bp HMGA2 2-8 and 600 bp HMGA2 2–5). Constructs containing two copies of let-7f complementary sequence were generated, with one construct containing a 27-nt sequence from lin41 (pmirGLO-let7) and one containing a shorter (s) 10-nt random sequence between the two copies of let-7f complementary sequence [pmirGLO-let7(s)]. Arrows show the let-7f interacting site. Underlined nucleotides show let-7f complementary sequence. **(B)** Schematic of the pmirGLO reporters assayed. Red rectangle indicates the reporter chosen for further experiments in the main article.
(TIF)

Figure S2 **Selection of luciferase reporter.** **(A)** The steady-state levels of let-7 in BG-1 and UCI-101 cells were measured by RT-qPCR analysis and represented relative to the levels of let-7 in a reference cell line derived from normal ovarian epithelium (HOSE-B cells), which were set as 1. Data are the means of two experiments yielding similar results. **(B,C)** Twenty-four hours after transfection of 100 nM pre-let-7f or Ctrl miRNA, BG-1 cells (B) or UCI-101 cells (C) were transfected with the reporters shown in Figure S1B. Twenty-four hours later, luciferase activity (FL/RL) was measured. Values shown are relative to Ctrl miRNA luciferase readings.
(TIF)

Figure S3 **Luciferase reporters psiCHECK2-POLE4 and psiCHECK2-MKK4 lacking let-7 sites are refractory to let-7 overexpression.** Forty-eight hours after transfection with 100 nM let-7 family precursors individually or Ctrl miRNA, BG-1 cells were transfected with luciferase reporters containing either *POLE4* 3′UTR or *MKK4* 3′UTR. Luciferase activity (RL/FL) was measured 24 h later and normalized to luciferase activity in Ctrl miRNA transfections. Data are the means of three independent experiments.
(TIF)

Figure S4 **GSK-3β silencing does not affect luciferase reporters psiCHECK2-POLE4 and psiCHECK2-MKK4.** Twenty-four hours after transfection with 100 nM Ctrl or GSK-3β siRNA, BG-1 cells were transfected with luciferase reporters psiCHECK2-POLE4 and psiCHECK2-MKK4. Luciferase activity (RL/FL) was measured 24 h later and normalized to Ctrl siRNA.
(TIF)

Figure S5 **Steady-state levels of let-7 in HCT116 (p53+/+) and HCT116 (p53−/−).** Let-7 levels in HCT116 cells were measured by RT-qPCR analysis and calculated relative to the levels of let-7 in a reference cell line derived from normal ovarian epithelium (HOSE-B cells), which were set as 1. Data are the means of two experiments yielding similar results.
(TIF)

Table S1 **Survey of let-7 levels using kinase inhibitor library and psiCHECK2-POLE4.** Five hours after transfection of psiCHECK2-POLE4, BG-1 cells were treated with the kinase inhibitor library and luciferase activity (RL/FL) was measured 24 h later. Inhibitor drugs triggering ‘Downregulated reporter activity’ (elevated let-7) were those yielding luciferase activities <0.6, while drugs triggering ‘Upregulated reporter activity’ yielded luciferase activities >1.8. All other drugs were classified as having ‘No effect on reporter activity’.
(TIF)

Table S2 **Survey of let-7 levels using kinase inhibitor library and psiCHECK2-MKK4.** Five hours after transfection of psiCHECK2-MKK4, BG-1 cells were treated with the kinase inhibitor library and luciferase activity (RL/FL) was measured 24 h later. Inhibitor drugs triggering ‘Downregulated reporter activity’ (elevated let-7) were those yielding luciferase activities <0.6, while drugs triggering ‘Upregulated reporter activity’ yielded luciferase activities >1.8. All other drugs were classified as having ‘No effect on reporter activity’.
(TIF)
