# Supplementary figures and images for: Novel MicroRNA Reporter Uncovers Repression of Let-7 by GSK-3β
Source: PLoS One. 2013 Jun 26;8(6):e66330. doi: 10.1371/journal.pone.0066330 (PMC3694080; doi:10.1371/journal.pone.0066330)

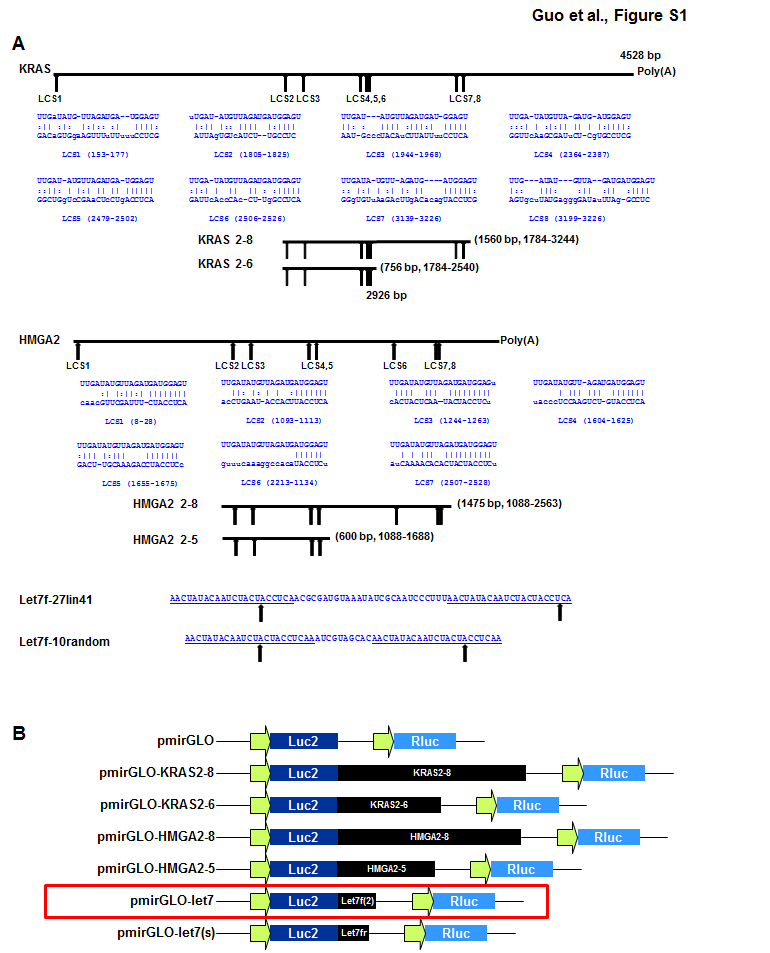

Supplement: Figure S1 — Construction of luciferase reporters. (A) The human KRAS 3′UTR has 8 let-7 complementary sequences (LCS) that can interact with let-7f, while the human HMGA2 3′UTR has 8 copies of LCS interacting with let-7f. The interactions are indicated (blue text). Various sequences were inserted into pmirGLO vector multiple cloning site, including two regions from human KRAS 3′UTR (1560 bp KRAS 2-8 and 756 bp KRAS 2–6), and two regions from human HMGA2 3′UTR (1475 bp HMGA2 2-8 and 600 bp HMGA2 2–5). Constructs containing two copies of let-7f complementary sequence were generated, with one construct containing a 27-nt sequence from lin41 (pmirGLO-let7) and one containing a shorter (s) 10-nt random sequence between the two copies of let-7f complementary sequence [pmirGLO-let7(s)]. Arrows show the let-7f interacting site. Underlined nucleotides show let-7f complementary sequence. (B) Schematic of the pmirGLO reporters assayed. Red rectangle indicates the reporter chosen for further experiments in the main article. (TIF) [file pone.0066330.s001.tif]

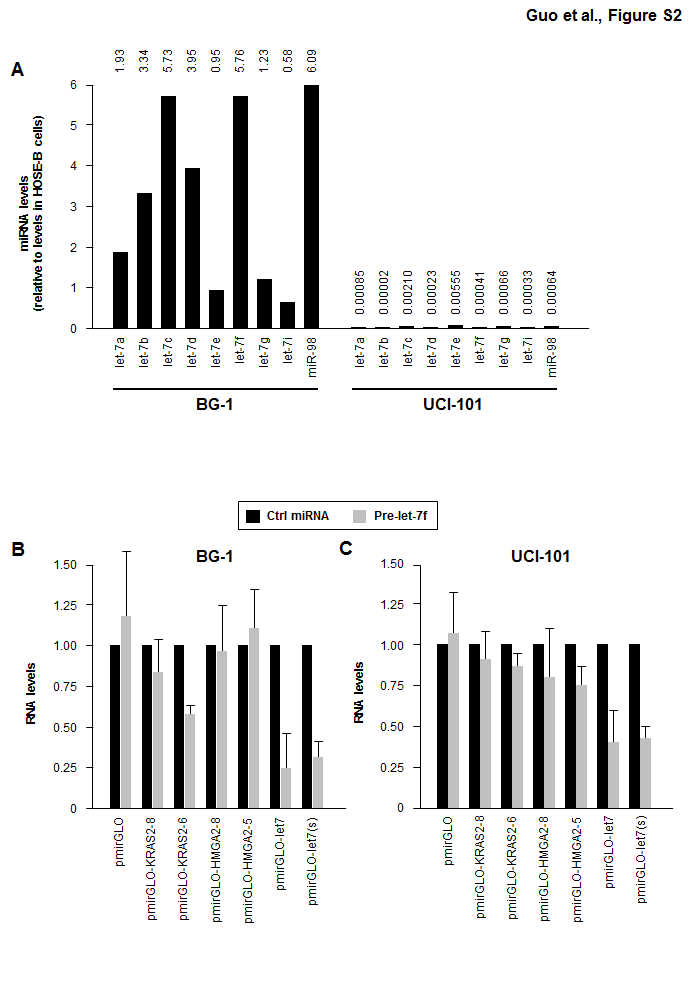

Supplement: Figure S2 — Selection of luciferase reporter. (A) The steady-state levels of let-7 in BG-1 and UCI-101 cells were measured by RT-qPCR analysis and represented relative to the levels of let-7 in a reference cell line derived from normal ovarian epithelium (HOSE-B cells), which were set as 1. Data are the means of two experiments yielding similar results. (B,C) Twenty-four hours after transfection of 100 nM pre-let-7f or Ctrl miRNA, BG-1 cells (B) or UCI-101 cells (C) were transfected with the reporters shown in Figure S1B. Twenty-four hours later, luciferase activity (FL/RL) was measured. Values shown are relative to Ctrl miRNA luciferase readings. (TIF) [file pone.0066330.s002.tif]

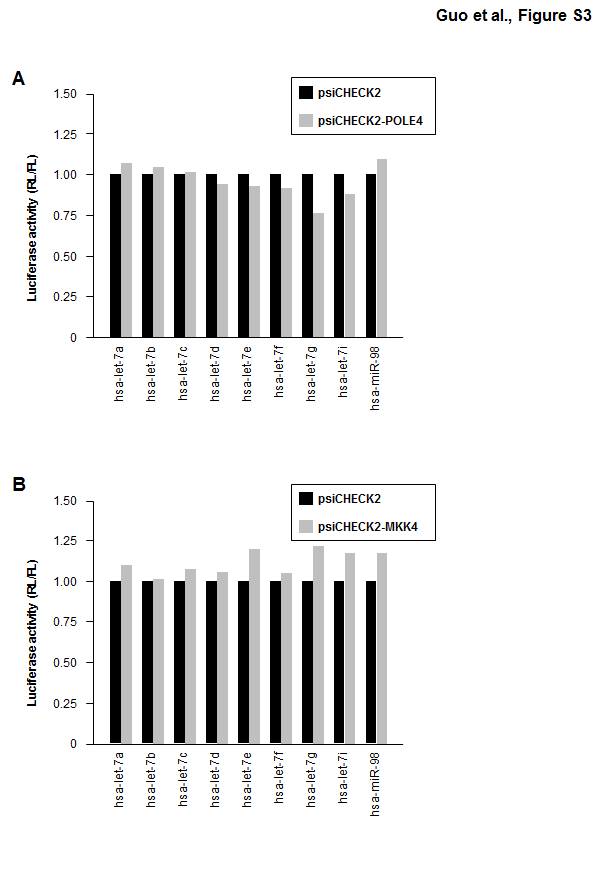

Supplement: Figure S3 — Luciferase reporters psiCHECK2-POLE4 and psiCHECK2-MKK4 lacking let-7 sites are refractory to let-7 overexpression. Forty-eight hours after transfection with 100 nM let-7 family precursors individually or Ctrl miRNA, BG-1 cells were transfected with luciferase reporters containing either POLE4 3′UTR or MKK4 3′UTR. Luciferase activity (RL/FL) was measured 24 h later and normalized to luciferase activity in Ctrl miRNA transfections. Data are the means of three independent experiments. (TIF) [file pone.0066330.s003.tif]

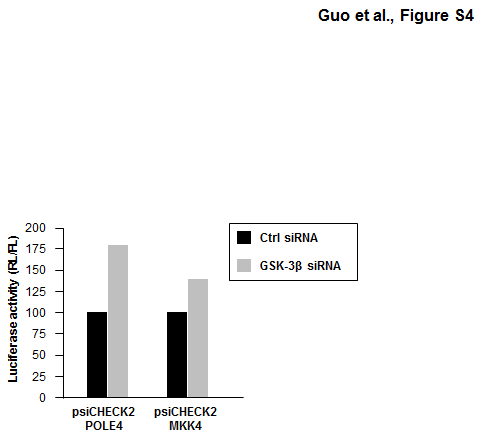

Supplement: Figure S4 — GSK-3β silencing does not affect luciferase reporters psiCHECK2-POLE4 and psiCHECK2-MKK4. Twenty-four hours after transfection with 100 nM Ctrl or GSK-3β siRNA, BG-1 cells were transfected with luciferase reporters psiCHECK2-POLE4 and psiCHECK2-MKK4. Luciferase activity (RL/FL) was measured 24 h later and normalized to Ctrl siRNA. (TIF) [file pone.0066330.s004.tif]

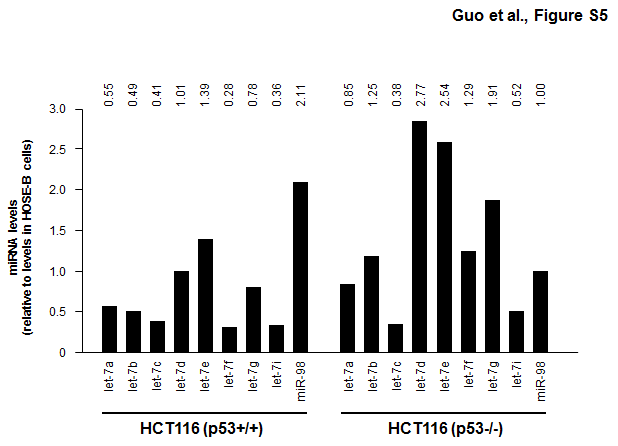

Supplement: Figure S5 — Steady-state levels of let-7 in HCT116 (p53+/+) and HCT116 (p53−/−). Let-7 levels in HCT116 cells were measured by RT-qPCR analysis and calculated relative to the levels of let-7 in a reference cell line derived from normal ovarian epithelium (HOSE-B cells), which were set as 1. Data are the means of two experiments yielding similar results. (TIF) [file pone.0066330.s005.tif]

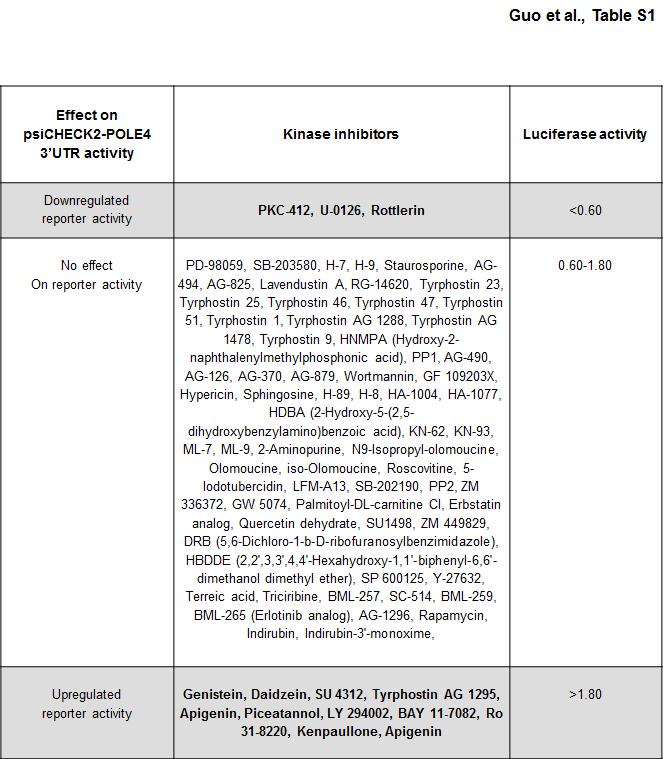

Supplement: Table S1 — Survey of let-7 levels using kinase inhibitor library and psiCHECK2-POLE4. Five hours after transfection of psiCHECK2-POLE4, BG-1 cells were treated with the kinase inhibitor library and luciferase activity (RL/FL) was measured 24 h later. Inhibitor drugs triggering ‘Downregulated reporter activity’ (elevated let-7) were those yielding luciferase activities <0.6, while drugs triggering ‘Upregulated reporter activity’ yielded luciferase activities >1.8. All other drugs were classified as having ‘No effect on reporter activity’. (TIF) [file pone.0066330.s006.tif]

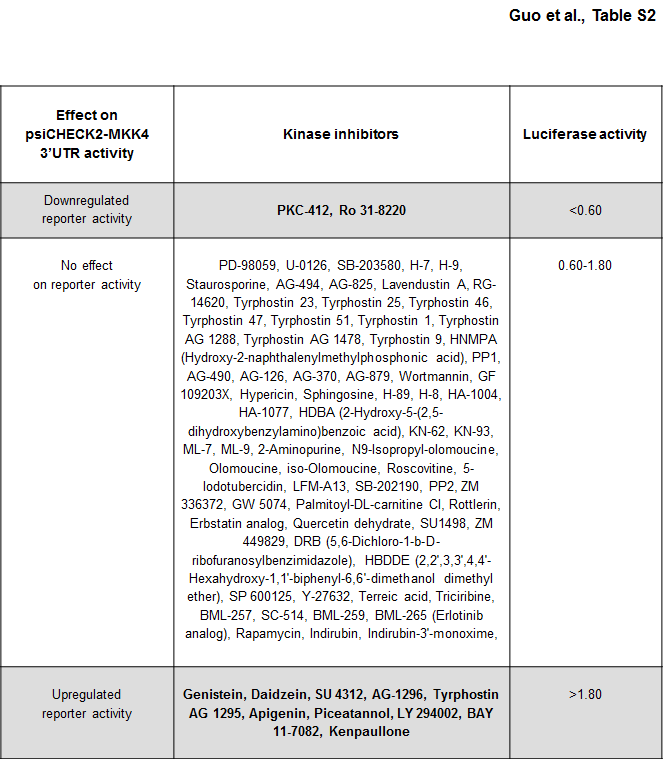

Supplement: Table S2 — Survey of let-7 levels using kinase inhibitor library and psiCHECK2-MKK4. Five hours after transfection of psiCHECK2-MKK4, BG-1 cells were treated with the kinase inhibitor library and luciferase activity (RL/FL) was measured 24 h later. Inhibitor drugs triggering ‘Downregulated reporter activity’ (elevated let-7) were those yielding luciferase activities <0.6, while drugs triggering ‘Upregulated reporter activity’ yielded luciferase activities >1.8. All other drugs were classified as having ‘No effect on reporter activity’. (TIF) [file pone.0066330.s007.tif]
